# Movement evaluation of front crawl swimming: Technical skill versus aesthetic quality

**DOI:** 10.1371/journal.pone.0184171

**Published:** 2017-09-08

**Authors:** Paola Zamparo, Stefano Carrara, Paola Cesari

**Affiliations:** Department of Neuroscience, Biomedicine and Movement Sciences, University of Verona, Verona, Italy; University of Bologna, ITALY

## Abstract

The study aim was to compare expert with non-expert swimmers’ rating of the aesthetic and technical qualities of front crawl in video-taped recordings of swimmers with low, middle, and high level proficiency. The results suggest that: i) observers’ experience affects their judgment: only the expert observers correctly rated the swimmers’ proficiency level; ii) evaluation of movement (technical and aesthetic scores) is correlated with the level of skill as expressed in the kinematics of the observed action (swimming speed, stroke frequency, and stroke length); iii) expert and non-expert observers use different strategies to rate the aesthetic and technical qualities of movement: equating the technical skill with the aesthetic quality is a general rule non-expert observers follow in the evaluation of human movement.

## Introduction

We addressed the question of whether the technical and aesthetic evaluation of an observed action can be attributed to specific kinematic characteristics of the movement observed and whether this depends on the level of skill in the movement that an evaluator has. Previous work has shown that actions become recognizable even when movements are presented as simple constellations of moving light points, underlying the human capability to “read” the kinematics of the movement observed [1]. This capability was further investigated in subsequent studies in which only experts in a specific sport were able to correctly anticipate the movements of the sport observed in a video recording [2–4].

It appears that evaluating the aesthetic component of an action might require the same categorization as that needed to evaluate its kinematics components. Interestingly though, when Scully [5] asked qualified gymnastic judges and naïve observers to rate the technical and aesthetical components in 30 balance beam routines, the naïve observers showed the highest correlations between the two movement components. Scully [5] suggested that this type of thinking would influence a typical naïve evaluation: “if the gymnast appears proficient, then she may also appear more aesthetic and vice versa”. Different is the scenario for qualified judges since, for them, a smoothly executed, aesthetically pleasing movement will not necessarily imply a technically efficient action. Following these observations, we recently conducted a study [6] evaluating Tai Chi performance and observed that: i) movement evaluation was positively correlated with the level of skill expressed in the kinematics of the observed action; ii) both expert and non-expert observers were able to discern good from poor performance; but iii) only the expert observers were able to discriminate the technical from the aesthetic components of the action evaluated.

Sport activities can be defined as either aesthetic or purposive [7]. A gymnastics beam routine can be considered a typical aesthetic sport since the aesthetic components are included in the overall evaluation. Though the expression of “aesthetic” components may not be a priority in Tai Chi, it does incorporate some features that could be perceived as aesthetically pleasing (e.g., fluency and rhythm of movement) [8]. Indeed, as previously found by Scully for gymnastics [5], we found a tight relationship between aesthetic and technical qualities in Tai Chi, as well as between these evaluations and the level of skill [6]. It therefore appeared interesting to further investigate the relationship between technical and aesthetic components in human movement in “pure purposive” sports to determine whether equating technical skill with aesthetic quality represents a general rule for the evaluation of human movement.

As defined by Best [7], typical purposive sports are sports in which “the aim, purpose or end can be specified independently of the manner of achieving it, as long as it conforms to the limits set by the rules or norms”; Best continues, stating that in such sports “certain moves or movements, indeed whole game or performances, can be considered from the aesthetic point of view; but that is a relatively unimportant aspect of the activity” [7]. Examples are athletics (the aim is to run faster, jump higher or throw an object further) or cyclic sports (e.g., running, cycling, swimming) where the purpose is to cover a given distance in the shortest time possible (to reach the highest speed). Swimming, the action investigated in the current study, is a typical purposive sport because, as pointed out by Best [7] “the aesthetic aspect is subordinate to the main purpose”. For this sport we can identify two main parameters that are an index of good performance/high technical skill: swimming speed (best swimmers are faster) and propelling efficiency (best swimmers are more efficient). Propelling efficiency (*E*_*p*_ indicates the capability of a swimmer to exert useful forces in water [9–11]. Since *E*_*p*_ tends to decrease when speed increases [12–13], it is also interesting to investigate this aspect at different paces (slow and fast).

In line with previous work in Tai Chi [6] and gymnastics [5], the hypotheses of this study were that: i) expert observers would be better able to distinguish good from poor swimming performance than non-expert observers, since the level of expertise in performing a sequence of movements correlates with the ability to judge the same movement when observed. As we are dealing with a mainly purposive sport, we expected that ii) technical scores would be higher than aesthetic scores and that iii) only the expert observers would be able to differentiate the technical from the aesthetic component of the action evaluated. Finally, iv) we expected that evaluation of movement would be correlated with the level of skill expressed in the kinematics of the observed action (e.g., with swimming speed and propelling efficiency).

## Materials and methods

### Biomechanics of front crawl (the basics)

The performance under analysis was front crawl swimming. In front crawl, propulsive forces are essentially generated by upper limb motion; the contribution of the lower limbs to propulsion (in terms of speed) is about 10% in this stroke [13]. In swimming, therefore, stroke frequency (*SF*) and stroke length (*SL*, the distance covered per stroke) are the main kinematic parameters that influence performance (*V* = *SF*^.^ *SL*). An increase in swimming speed is mainly obtained by increasing *SF*, whereas *SL* is maximal at slow swimming speeds and tends to decrease as speed increases [12–13]. Moreover, as shown by Craig and Pendergast [14], for a given speed, *SF* is lower for elite athletes as compared to less experienced swimmers (i.e., at the same *SF*, elite athletes swim faster and with a larger *SL*). Propelling efficiency changes as a function of speed in a manner similar to *SL* [12, 13, 15]: efficiency is greater the slower the speed and the more experienced the swimmer.

### Participants

Thirty male participants were recruited and divided into two groups: 15 were swimming instructors with lengthy swimming experience (10.3 ± 2.0 years), hereafter defined as expert observers (EO), and 15 had little swimming experience (1.1 ± 0.9 years) and were defined as non-expert observers (NEO) (see Table 1).

**Table 1:** Observers’ characteristics (data are means ± SE).

Observers	EO (N = 15)	NEO (N = 15)
Age (years)	35.5 ± 2.9	30.1 ± 1.3
Experience (years)	10.3 ± 2.0	1.1 ± 0.9 *

**Footnote:** Expert observers (EO); non-expert observers (NEO).

* p < 0.001.

There were no differences in age between the two groups (unpaired t-test, p > 0.1). Table 1 reports the years of experience the expert observers had as instructors; 10 of them were also former competitive swimmers and all practiced swimming regularly. The non-expert observers were postgraduate and Ph.D students recruited at the local university; they had either no or only minimal swimming experience (nine had learned to swim as a child and now swim only occasionally, and six had never learned how to swim). All participants received written and oral instructions before the study began and gave their informed written consent. The Institutional Review Board (Ethics Committee of the Department of Neuroscience, Biomedicine and Movement Sciences, University of Verona, Italy) approved the study protocol; the study was conducted according to the principles expressed in the Declaration of Helsinki.

### Stimuli: Video clip preparation

Video clips were prepared by recording front crawl swims performed by 27 male master swimmers. All swimmers wore a traditional swimsuit and were video recorded via an underwater remote camera video system (SeaViewer Cameras, Inc, Tampa, FL, USA, 50 fps) positioned at a depth of 0.5 m below the waterline and frontally to the swimmer.

Ten of the 27 swimmers were qualified as “high-level performers” ([HLP] 15.7 ± 3.8 years of swimming practice), ten as “middle-level performers” ([MLP] 9.6 ± 2.2 years of swimming practice), and seven as “low-level performers” ([LLP] 1.6 ± 0.3 years of swimming practice). The swimmers received written and oral instructions before the study began and gave their informed written consent to participating in the experimental procedure. There were no differences in body mass, stature, age, and number of training hours per week between the swimmers (one way ANOVA, 0.190<p>0.684), whereas years of experience differed between the three groups (p = 0.009); the post hoc Tukey HSD test revealed a significant difference between the HLP and the LLP group (p = 0.007), whereas the MLP group was similar to both the HLP (p = 0.262) and the LLP group (p = 0.155) (Table 2).

**Table 2:** Swimmers’ characteristics (data are means ± SE).

Characteristic	HLP (N = 10)	MLP (N = 10)	LLP (N = 7)
Body mass (kg)	75.1 ± 2.4	76.3 ± 3.2	72.5 ± 3.3
Stature (cm)	180 ± 2.4	180 ± 1.5	175 ± 1.3
Age (years)	31.8 ± 2.4	34.9 ± 3.5	37.9 ± 3.7
Experience (years)	15.7 ± 3.8	9.6 ± 2.2	1.6 ± 0.3 *
Training (h/week)	3.2 ± 0.1	2.6 ± 0.2	2.6 ± 0.2

**Footnote:** Level of performance: HLP: high-level performers; MLP: middle-level performers; LLP: low-level performers

* p < 0.01: LLP ≠ HLP.

### Kinematic analysis

The three groups of swimmers were also categorized by means of kinematics analysis of their individual swimming performance. They were asked to swim the front crawl at six incremental self-selected speeds, from slow to fast, with at least 3 minutes rest in between trials. The experiments were conducted in a 25-m indoor swimming pool; all parameters were assessed in the central 10-m of the lane to avoid the influence of push-off start and finish. The actual speed (clean swimming speed: *V*, m^.^ s^-1^) was measured from the time taken to cover the middle 10 m, during which the average stroke frequency (*SF*, Hz) was computed from the time taken to complete a given number of strokes. The distance covered per stroke (stroke length, *SL*, m) was calculated by dividing the average speed by the corresponding stroke frequency (*SL = V/SF*). Arm-stroke efficiency (*η*_*P*_) was calculated according to the model proposed by Zamparo and co-workers [12] in which the upper arm is considered a rigid segment of length *l* rotating at a constant angular speed *(ω* = 2π *SF*) around the shoulder, as follows:

$$E_{p}=\left(\frac{V∙0.9}{2\pi SFl}\right)∙\frac{2}{\pi }$$

where *V* is the average swimming speed (multiplied by 0.9 to take into account that the arms contribute about 90% to propulsion in this stroke [13]), *SF* is the stroke frequency, and *l* is the shoulder-to-hand distance; the latter was calculated as described below.

An underwater video camera system (SeaViewer, Tampa, FL, USA, 50 fps) was positioned 0.5-m below the surface and frontally to the swimmer’s direction. Video clips were digitized using a commercial software package (Twin Pro, SIMI, G) and the elbow angle was measured at the end of the in-sweep phase (when the plane of the arm and forearm is perpendicular to the optical axis of the camera) for the right and left sides and for different arm strokes. The average elbow angle was then used to calculate *l* by trigonometry by knowing the arm and forearm lengths (measured with a meter tape to the nearest 0.01 cm). The swimmers were instructed to hold their breath during the last 10–15 m of the lane (where the elbow angle was measured) since adjustments in body position in water necessary to perform this action do, indeed, affect the measurement of this parameter.

Only data referring to the first (slow) and last (fast) pace where utilized for further analysis. The observers watched the video clips (27 x 2) and rated the technical and aesthetic qualities of the swimmers’ performance as described in detail below (Procedure).

Swimming speed (*V*), stroke frequency (*SF*, Hz), stroke length (*SL*, m), and propelling efficiency (*η*_*P*_) at the two swimming paces (slow and fast) are reported in Table 3.

**Table 3:** Kinematic data at slow and fast swimming pace (data are means ± SE).

Swimming pace		HLP (N = 10)	MLP (N = 10)	LLP (N = 7)
slow	V (m^.^s^-1^)	0.99 ± 0.02	0.89 ± 0.03	0.78 ± 0.03
	SF (Hz)	0.42 ± 0.02	0.47 ± 0.02	0.78 ± 0.02
	SL (m)	2.39 ± 0.17	1.93 ± 0.06	1.82 ± 0.13
	E_p_	0.36 ± 0.03	0.29 ± 0.01	0.30 ± 0.03
fast	V (m^.^s^-1^)	1.58 ± 0.03	1.39 ± 0.03	1.03 ± 0.04
	SF (Hz)	0.82 ± 0.03	0.79 ± 0.03	0.64 ± 0.03
	SL (m)	1.96 ± 0.08	1.76 ± 0.05	1.64 ± 0.11
	E_p_	0.30 ± 0.01	0.26 ± 0.01	0.27 ± 0.02

**Footnote:** Level of performance: HLP: high-level performers; MLP: middle-level performers; LLP: low-level performers; *V*: swimming speed; *SF*: stroke frequency; *SL*: stroke length; *E*_*p*_: propelling efficiency.

ANOVA for repeated measures of two levels for pace (slow, fast) X three levels for group (HLP, MLP, LLP) was performed with Bonferroni adjustments for each of the parameters listed in Table 3. The three groups differed significantly in swimming speed (F_(1,24)_ = 54.200, p < 0.001). Pairwise comparisons showed that the HLP were faster than the MLP that, in turn, were faster than the LLP (p < 0.001 for all comparisons). The main factor speed was also significant (F_(1,24)_ = 514.413, p < 0.001) as was the interaction pace X group (p < 0.001). A significant difference between the two paces was observed within each group.

The three groups differed significantly in stroke frequency (F_(1,24)_ = 4.496, p = 0.022). Pairwise comparisons showed that each group differed from each other at the slow pace, whereas at the fast pace the stroke frequency was higher for the HLP than the LLP (p < 0.001); there was no significant difference in stroke frequency between the MLP and the HLP or the LLP (p > 0.05). The main factor for stroke frequency was significant (F_(1,24)_ = 110.905, p < 0.001), along with the interaction frequency X group (p < 0.001). Stroke frequency was higher at fast pace than at slow pace and this difference was present within each group.

The three groups differed significantly in stroke length (F_(1,24)_ = 5.858, p = 0.008). Pairwise comparisons showed that stroke length was larger for the HLP than either the MLP or the LLP (p = 0.045 and 0.013, respectively), no significant difference in stroke length between the MLP and the LLP was noted (p > 0.05). The main factor stroke length was significant (F_(1,24)_ = 23.316, p < 0.001): stroke length was larger at slow than at fast pace. The interaction stroke length X group was not significant (p > 0.05).

The three groups differed significantly in propelling efficiency (F_(1,24)_ = 3.945, p = 0.033). Pairwise comparisons showed that propelling efficiency was higher for the HLP than the MLP (p = 0.044). No other differences were observed. The main factor for propelling efficiency was significant (F_(1,24)_ = 24.046, p < 0.001): propelling efficiency was greater at slow than at fast pace. The interaction efficiency X group was not significant (p > 0.05).

**Fig 1:**
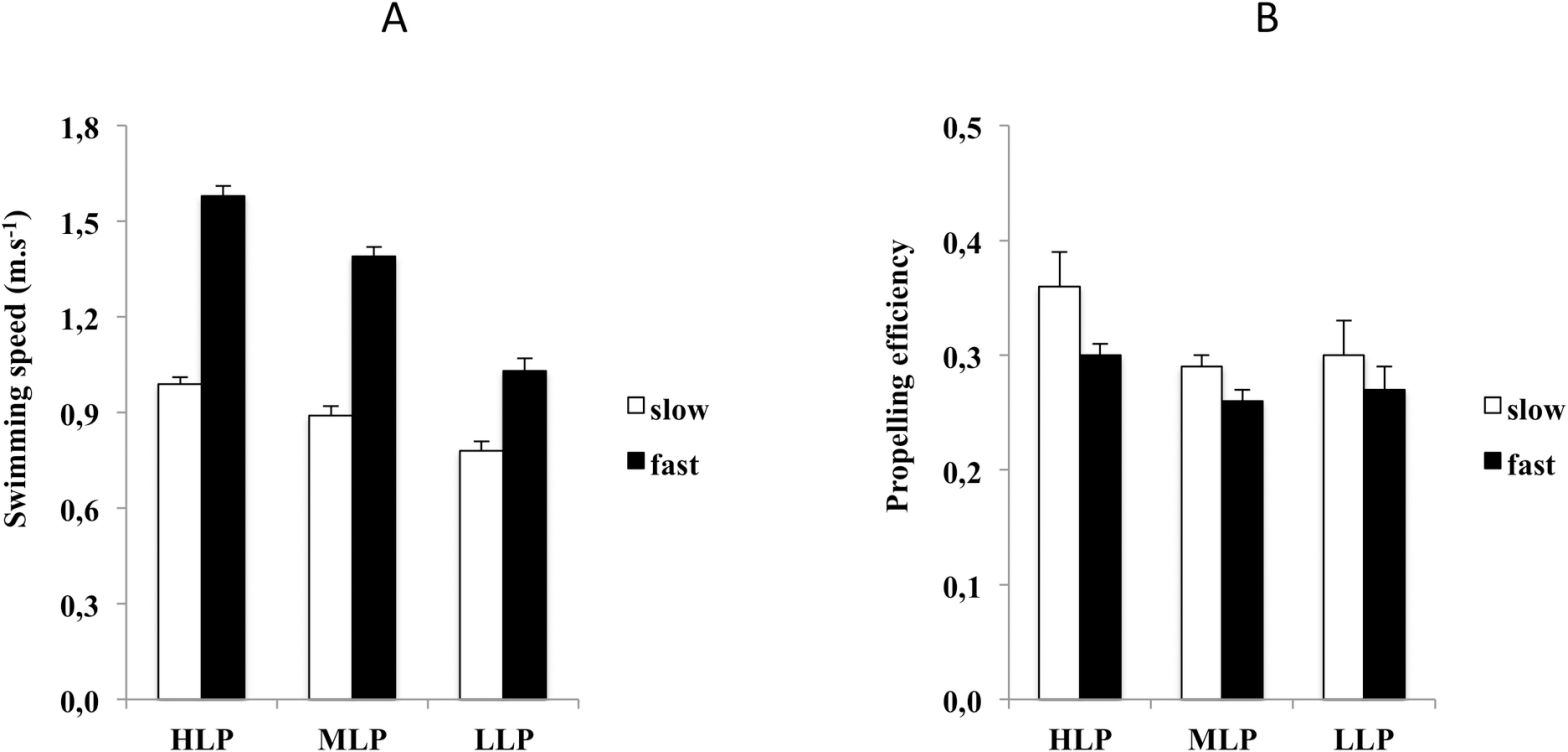
A and B: Swimming speed (m^.^s^-1^) (Panel A) and propelling efficiency (Panel B) in the three groups of swimmers ([HLP] high-level performers; [MLP] middle-level performers; [LLP] low-level performers) at slow (white columns) and fast (black columns) pace. Data are means ± SE.

Summarizing, an increase in swimming pace (from slow to fast) was associated with an increase in *V* and *SF* and a decrease in *SL* and *E*_*p*_. Moreover, longer swimming experience was associated with higher values of *V*, *SL*, and *E*_*p*_ at both slow and fast paces, indicating that with more swimming practice, movements become faster (larger *V*), “wider” (longer *SL*), and more efficient (greater *E*_*p*_) (Fig 1A and 1B).

## Procedure

For the observational task, the video clips were cut using appropriate software (Windows Movie Maker) and then evaluated with E-prime V2.0 (SP1) software (Psychology Software Tools, Inc., Pittsburgh, PA, USA). The average duration of each video clip was about 4 s. The observers watched the video clips and rated swimmer performance on a visual analogue scale (VAS) [16], where 0 indicates poor performance and 10 excellent performance. They were also asked to rate with two separate VAS scores i) the level of skill and ii) the level of beauty of the movement observed.

Before data collection, the observers were instructed on how to use the VAS scale and were shown two selected video clips (at fast pace), one taken of an excellent swimmer (HLP with 20 years of practice) and one of a low-level swimmer (LLP with 1 year of practice) (see also [9]). As suggested by Scully [5], observer judgement was based on “a priori presentation of a standard”. The observers received no feedback while watching the two video clips, the only aim being to illustrate the two ends of the range of values for rating swimming performance on the VAS scale. The order of presentation of the video clips was randomized across participants (E-prime V2.0 (SP1)). The two video clips shown before the experiments are reported as supporting information (S1 Video: a good swimming performance; S2 Video: a poor swimming performance). A PLOS consent form was signed by these participants.

## Data analysis

The VAS data were compared using ANOVA with repeated measurements (3 X 2 X 2 X 2), considering the three levels of performance (HLP, MLP, and LLP), the two paces (slow and fast), and the two components of movement qualities (Technical [T] and Aesthetic [A]) as within-subject factors, and the two observer groups (experts [EO] and non-experts [NEO]) as between-subjects factor. Post-hoc comparisons were performed using t-tests with the Bonferroni correction for multiple comparisons when required. Linear regressions were computed using the method of least squares to investigate the relationship between VAS data and kinematic variables; the correlation coefficient (*R*) was used to indicate the goodness of fit. In all analyses, the significance level was set at *p* < 0.050. Data are reported as means ± SE.

## Results

Table 4 presents the descriptive statistics of the observed variables.

**Table 4:** VAS score (0–10) at slow and fast swimming pace (data are means ± SE).

		T (slow)	A (slow)	T (fast)	A (fast)
EO	HLP	5.86 ± 0.34	5.44 ± 0.34	5.65 ± 0.30	4.89 ± 0.38
	MLP	4.84 ± 0.36	4.40 ± 0.40	5.09 ± 0.34	4.53 ± 0.25
	LLP	2.62 ± 0.44	2.13 ± 0.42	3.32 ± 0.50	2.75 ± 0.46
NEO	HLP	4.48 ± 0.33	4.11 ± 0.34	2.98 ± 0.37	2.56 ± 0.33
	MLP	4.15 ± 0.32	3.67 ± 0.34	4.02 ± 0.26	3.61 ± 0.33
	LLP	4.64 ± 0.38	4.15 ± 0.34	2.46 ± 0.45	1.91 ± 0.48

**Footnote:** EO: expert observers; NEO: non-expert observers; HLP: high-level performance; MLP: middle-level performance; LLP: low-level performance; T: technical qualities; A: aesthetic qualities.

A significant difference (main effect) was noted between the two observer groups (F_(2,28)_ = 5.426, p < 0.027): the expert observers (EO) rated overall swimmer performance generally higher than the non-expert observers (NEO), and both groups gave higher VAS scores to the technical than to the aesthetic qualities. ANOVA showed (main effect) that technical and aesthetical qualities were evaluated differently (F_(1,28)_ = 26.404, p < 0.001), with higher scores awarded for the technical qualities.

**Fig 2:**
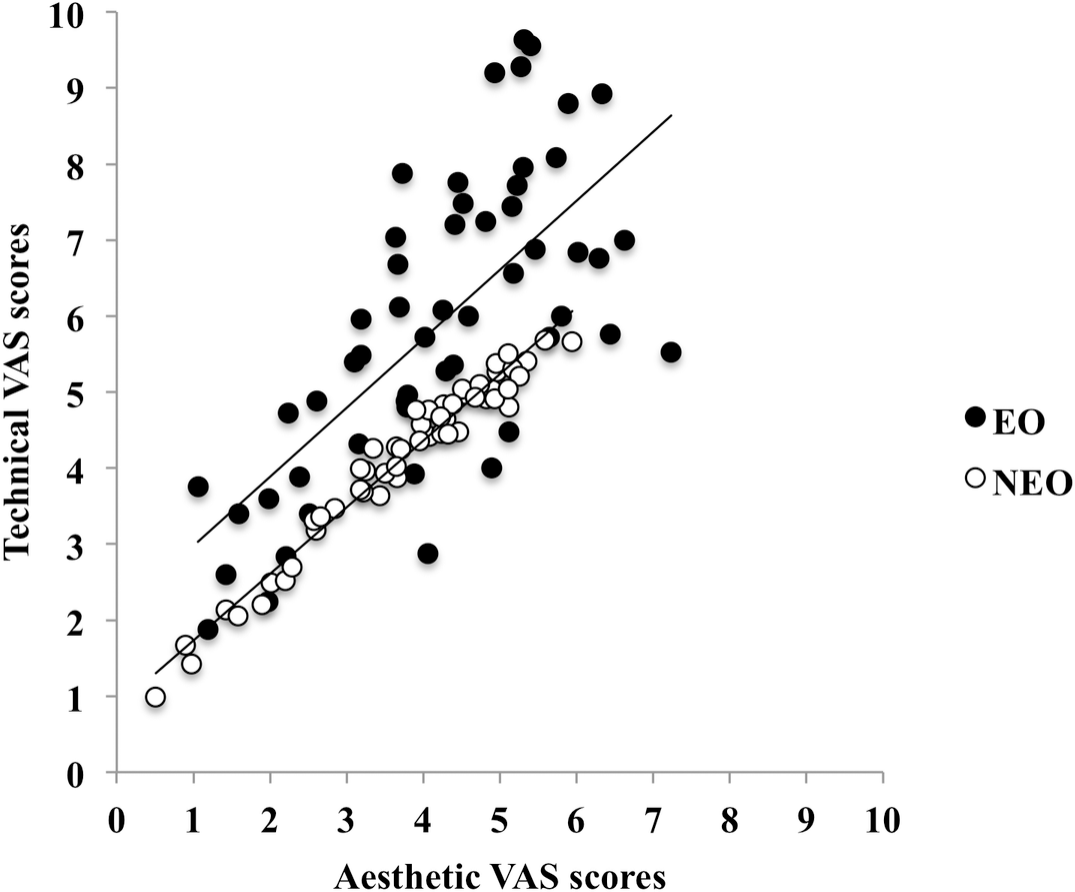
Technical (T) vs. aesthetic (A) qualities: VAS scores given to all swimmers (at slow and fast pace) by expert ([EO] full dots) and non-expert ([NEO] open dots) observers.

These two main effects are clearly evident in Fig 2, which shows the relationship between technical (T) and aesthetic (A) qualities as rated by both observer groups for all swimmers and at both paces; these relationships are well described by the following linear regression: for expert observers (EO): T = 2.07 + 0.908^.^ A, N = 54, R^2^ = 0.482, p < 0.001; and for non-expert observers (NEO): T = 0.8557 + 0.8764^.^ A, N = 54, R^2^ = 0.960, p < 0.001. These equations indicate that technical and aesthetic qualities are strictly related in swimming; as compared to the EO, the regression is closer to the identity line for the NEO and with a larger coefficient of determination (lower scatter).

**Fig 3:**
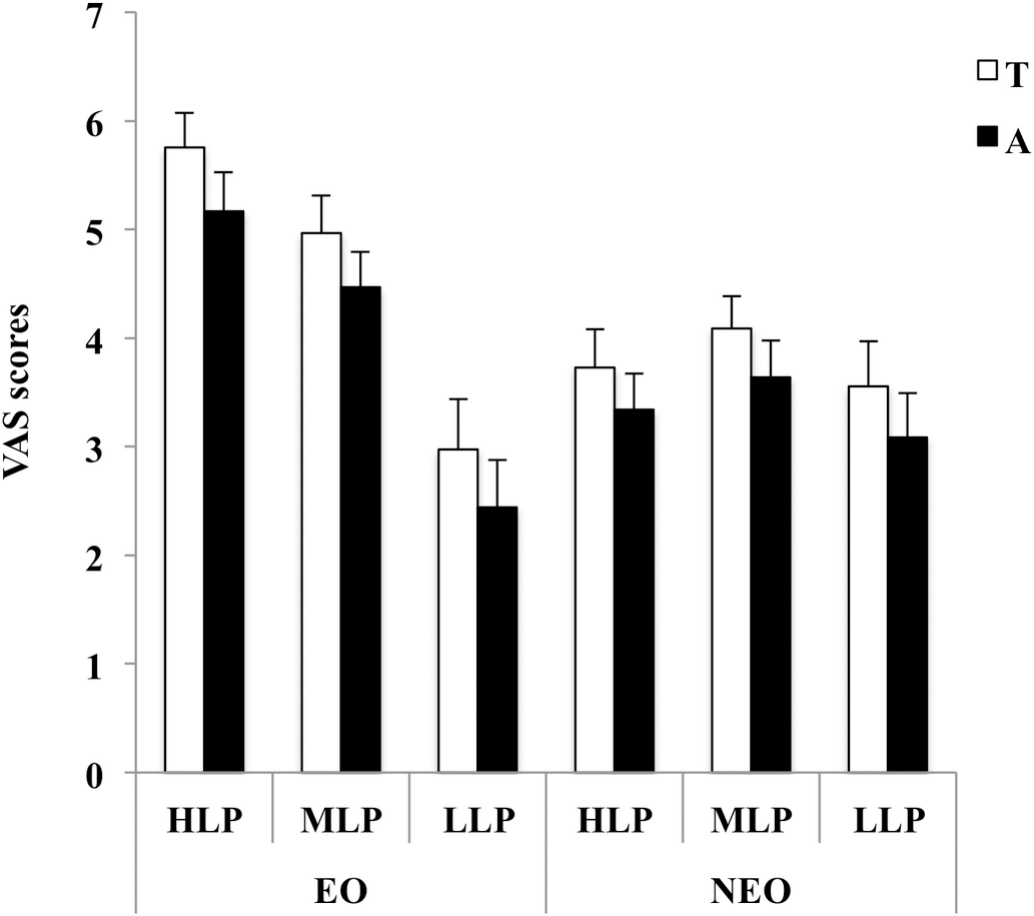
VAS scores (means ± SE) for technical ([T] white columns) and aesthetic ([A] black columns) qualities given by the expert (EO) and non-expert (NEO) observers to the swimmers ([HLP] high-level performers; [MLP] middle-level performers; [LLP] low-level performers). Values measured at slow and fast pace were merged.

Fig 3 presents the VAS scores given for technical (white columns, T) and aesthetic (grey columns, A) (average of slow and fast pace) qualities by the two observer groups: the expert observers “correctly” scored the swimmers from best to worst, whereas the non-expert observers gave the same scores to all swimmers.

The three levels of swimmer performance (high, middle, low) were recognized as differing from one another (F_(2,56)_ = 103.641, p < 0.001), along with significant interactions: performance level X velocity, performance level X observer group and performance level X velocity X observer group (p < 0.050 in all cases). Higher scores were given for HLP and MLP than for LLP (p < 0.001). At slow velocity, the three performance levels were easily distinguishable (all p < 0.001), whereas at fast velocity the HLP was recognized as differing from both the MLP and the LLP (p<0.001), the latter two of which, however, were indistinguishable from one another (p > 0.05). Expert observers clearly rated each performance level (p < 0.001 in all cases, Fig 3) and they were able to do so for both velocities. The non-expert observers were able to distinguish HLP from MLP and LLP but not between MLP and LLP (p> 0.05), generally when they were judging performance at slow velocity.

**Fig 4:**
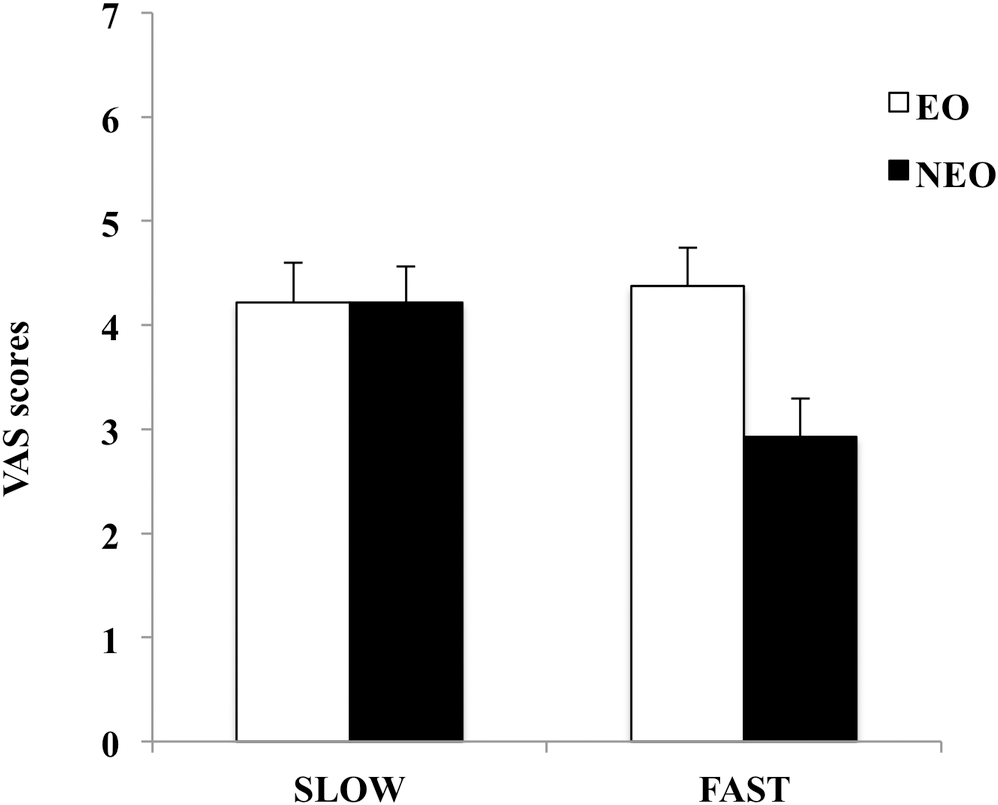
VAS scores (means ± SE) given by expert ([EO] white columns) and non-expert ([NEO] black columns) observers for performance at slow and fast pace. The values are the grand average of scores for technical and aesthetic qualities for all swimmers (HLP, MLP, and LLP).

Finally, the observers rated the two swimming paces differently (F_(1,28)_ = 15.102, p < 0.001): higher scores were given to swimming at slow than at fast pace (4.22 vs. 3.65). The average VAS score (both technical and aesthetic qualities for all three swimming proficiency levels) is reported in Fig 4. The interaction velocity X group (F_(1,28)_ = 24.950, p < 0.001) showed that these differences were attributable to the non-expert observers who tended to rate performance lower at fast pace (p < 0.000), whereas the expert observers rated performance at slow and fast pace in similar manner (p = 0.440).

### Correlation between VAS scores and movement kinematics

**Fig 5:**
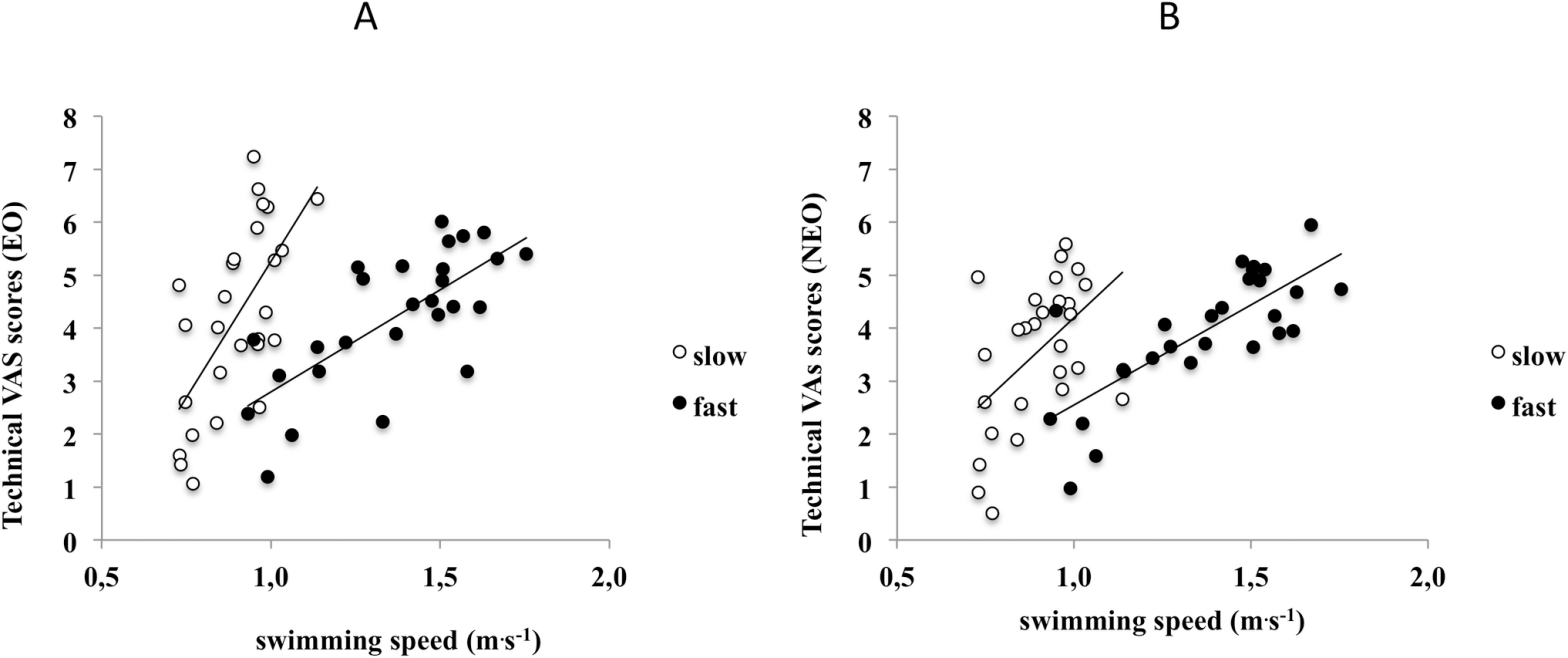
A and B: Relationship between VAS technical component scores and swimming speed (m^,^s^-1^) at slow (open dots) and fast (full dots) pace for expert observers (Panel A) and non-expert observers (Panel B).

Fig 5 reports the technical VAS scores as a function of swimming speed (*V*) at slow (open dots) and fast (full dots) pace. The upper panel refers to the EO and the lower panel to the NEO. A significant correlation was found between the kinematics of the action (swimming speed, in this case) and the technical evaluation of the action observed, and that this relationship has a different slope at the two paces (i. e., is larger at the slow pace). Similar results were obtained for the aesthetic component scores: a significant relationship was found between the VAS aesthetic scores and swimming speed both at slow and fast pace and for both observer groups (N = 27, 0.49<R>0.79, 0.01<p> 0.001 in all cases).

Significant relationships were found also between stroke frequency (*SF*) and VAS technical and aesthetic component scores at both paces, but only for the NEO (N = 27, 0.39<R> 0.63, 0.05<p> 0.001), and between stroke length (*SL*) and VAS aesthetic scores at slow pace for both observer groups (N = 27, 0.44<R>0.47, p < 0.05). No relationship was observed between propelling efficiency and VAS scores, either technical or aesthetic, for either observer group or either swimming pace.

## Discussion

We compared expert and non-expert observers in their ability to rate the technical and aesthetic components of video-recorded swimming performances. This study builds on a previous one in which Thai Chi, a martial art and non-purposive sport, was evaluated; in the present study we examined swimming, considered a pure-purposive sport.

As expected, and as previously found for Thai Chi performance [6] and gymnastic beam routine [5], both the expert and non-expert observer groups alike rated the technical components higher when evaluating swimming performance. No interaction with the observer group was found, indicating that the technical component was considered highly relevant no matter the observer’s level of experience with swimming.

When we regressed the technical component scores as a function of the aesthetic component scores for the observer groups separately, we found that the coefficient of correlation was highly significant for the non-expert observers and that the regression was very close to the identity line. Differently, the variability for the expert observers was more pronounced. This suggests that a technically well-performed action implies an aesthetically pleasing movement for the non-expert observers. Reformulating Scully’s words, it seems that for non-expert observers “if the swimmer appears proficient, then he or she may also appear more aesthetically appealing and vice versa”. As Scully points out further [5], the fact that non-expert observers essentially equate technical and aesthetic scores “may be due to their uncertainty as to the appropriate kinematics”. On the other hand, the positive correlation between the technical and aesthetic component scores given by the expert observers, although moderate as compared to the non-expert observers, supports the idea that “they found a relatively direct relationship between technical execution and aesthetic quality of performance”. This seems a common finding shared by both aesthetic and purposive sports.

As reported in our previous work, we found that scores were given based on the level of performance observed: higher scores for better performance and lower scores for poorer performance. This distinction was clearer for the expert observers, whereas the non-experts were not equally able to distinguish among the three performance levels. This difference supports the idea that being an expert evaluator/performer in a specific sport (all expert observers were highly experienced swimmers) will allow for better recognition of the components of an action [2–4].

This finding contrasts with our previous study on Tai Chi [6] in which both observer groups, expert and non-expert, were able to discern good from mediocre performance. Possible explanations are: i) the movement analyzed in the present study was the arm stroke (cyclically repeated) and only a few cycles could be observed while the swimmer was approaching the camera from the front, and ii) the video clip duration was much shorter in this study than in the previous one (4 s vs. 30 s), leaving fewer kinematic clues available to the observers (in Tai Chi more complex, and different, movements were under analysis). These cues were, however, sufficient for the expert observers to correctly score the three levels of proficiency. When asked about which kinematic clues they were observing, both observer groups reported that they were observing body alignment, joint position, point of hand entry in water, and hand path in the underwater/pulling phase; the differences between expert and non expert observers seem to be attributable to the non experts “uncertainty as to the appropriate kinematics”.

A slow pace was rated higher than a fast one, and a main effect was present. This might appear a bit counterintuitive since the best swimmer is by definition the fastest. To better understand the meaning of this result, we examined the significant interaction of velocity X observer group and found that the preference for slow pace was mainly due to the non-expert observers, while the expert observers were able to equally distinguish good from low performance when presented at slow or fast speed. Again, this effect for the non-expert observers could be attributed to “their uncertainty as to the appropriate kinematics”.

A strong relationship was found between aesthetic/technical component scores and swimming speed at both fast and slow pace, whereas no relationship was observed between propelling efficiency and either observer group. Therefore, in the trade-off between swimming speed (the greater the faster the pace) and propelling efficiency (the lower the faster the pace), the former “weights more” probably because the differences in speed among swimmers of different proficiency levels are larger than their differences in propelling efficiency. This seems to suggest that propelling efficiency is not a directly available kinematic clue, even if it is related to directly available clues such as speed and stroke frequency (see Equation in the Kinematic Analysis).

## Conclusions

Our findings suggest that in purposive and aesthetic sports alike: i) the observer’s level of motor experience affects their judgment, with the implication that being expert in a specific sport allows for the correct evaluation of the level of expertise; ii) movement evaluation is correlated with the level of skill expressed in the kinematics of the observed action; and iii) expert and non-expert observers use different strategies to rate the aesthetic and technical qualities of a movement. Equating technical skill with aesthetic quality seems a general rule for the evaluation of human movement in both purposive and aesthetic sports, and particularly so for non-expert observers.

## Supporting information

S1 Video A good swimming performance (HLP) at fast speed (AVI). (AVI)

S2 Video A poor swimming performance (LLP) at fast speed (AVI). (AVI)
